# Mapping of plasmonic resonances in nanotriangles

**DOI:** 10.3762/bjnano.4.66

**Published:** 2013-09-30

**Authors:** Simon Dickreuter, Julia Gleixner, Andreas Kolloch, Johannes Boneberg, Elke Scheer, Paul Leiderer

**Affiliations:** 1 Physics Department, University of Konstanz, Universitätsstraße 10, 78464 Konstanz, Germany

**Keywords:** ablation, FDTD simulations, field enhancement, nanotriangles, near field, surface plasmons

## Abstract

Plasmonic resonances in metallic nano-triangles have been investigated by irradiating these structures with short laser pulses and imaging the resulting ablation and melting patterns. The triangular gold structures were prepared on Si substrates and had a thickness of 40 nm and a side length of ca. 500 nm. Irradiation was carried out with single femtosecond and picosecond laser pulses at a wavelength of 800 nm, which excited higher order plasmon modes in these triangles. The ablation distribution as well as the local melting of small parts of the nanostructures reflect the regions of large near-field enhancement. The observed patterns are reproduced in great detail by FDTD simulations with a 3-dimensional model, provided that the calculations are not based on idealized, but on realistic structures. In this realistic model, details like the exact shape of the triangle edges and the dielectric environment of the structures are taken into account. The experimental numbers found for the field enhancement are typically somewhat smaller than the calculated ones. The results demonstrate the caveats for FDTD simulations and the potential and the limitations of “near field photography” by local ablation and melting for the mapping of complex plasmon fields and their applications.

## Introduction

Considering classical optics, light cannot be focused to a scale much smaller than half its wavelength. This phenomenon, commonly known as “diffraction limit”, represents a major obstacle in modern nanotechnology, as it prevents the use of well-established optical methods in the preparation of structures on the lower nanometer scale or the integration of optical parts into nanodevices. The diffraction limit can be overcome, however, by making use of the optical near fields of nanostructures, in particular metallic nanoparticles, which display pronounced plasmon resonances. These highly localized near fields of plasmonic particles have been demonstrated to be a very efficient tool for nanomachining [1], optical pumping of nanoscale objects such as quantum dots [2], surface enhanced Raman scattering [3–4] and extreme light confinement for nonlinear effects [5–7].

While the basic principles of computing plasmonic resonances are well understood (i.e., by using Maxwell´s equations on small objects), it has proven to be difficult to predict the field distribution for a given nanoscopic object. Two main factors are complicating this task: Firstly, solving Maxwell´s equations for a system more complex than a simple geometric object requires a certain amount of simplification or a careful numerical treatment. Commonly used numerical simulation methods are, for example, discrete dipole approximation (DDA) or finite-difference in the time-domain (FDTD). Secondly, the outcome of these simulations has to be compared to a measurement of the field distribution. Since the field enhancement can be highly confined, direct probing of the field distribution is rather challenging. Experimental approaches to visualize the near field include techniques like scanning nearfield optical microscopy (SNOM), in which a fine tip consisting of a thin optical fiber coated with metal is scanned across the sample. This probe can either be used to illuminate a small volume of the sample or to collect light from the near field of a sample, which is illuminated conventionally [8–10]. A related approach is to use a very fine metal or dielectrical tip as a scatterer in the vicinity of the optical near field of the sample [11–13]. Another method for mapping near fields is photoemission electron microscopy (PEEM), in which photoelectrons emitted from the plasmonic structure out of regions of high field enhancement are imaged in an electron microscope [14–15].

Roughly a decade ago, several techniques with ex-situ analysis have been developed, which can be considered as types of “near-field photography”. In these approaches, the near field is depicted in a matrix on which the scattering particle is placed or which encloses the particle itself [16–23]. These techniques share the common advantage that the resolution is believed to be limited mainly by the read-out process. As it is done after irradiation, highly advanced standard high-resolution microscopy methods can be used. Direct nanoscale ablation is a relatively straight-forward near-field photography technique that was demonstrated to depict the near field of nanostructures in great detail [21].

In this work, we describe our results for two techniques, which we have used to quantify optical near fields of plasmonic nano-triangles: on the one hand near-field photography by direct ablation of the substrate (silicon), on the other hand a new method, which utilizes the local melting of the gold nanostructures at sites of high field enhancement. These two techniques are variations of the same methods: The substrates carrying the nanostructures are irradiated with pulsed laser light. The ablation experiments are realized by using irradiation with femtosecond laser pulses, i.e., pulses shorter than the internal heat diffusion times. When using pico- and nanosecond pulses instead, the heat diffusion results in controlled melting of the nanostructures. The findings will be compared to the results of FDTD simulations, and also the limitations of the two techniques will be discussed. Finally we discuss the field-enhancing effects of the nano-triangles by comparison to equivalent experiments carried out on bare substrates. (We note that the enhancement factors discussed here do not refer to the electric field *E*, but to the field intensity, i.e., *E*^2^.) Triangles were chosen here instead of spheres or rods studied in other recent investigations [1,24–26], because they have sharp tips where the localization of the field enhancement can be particularly pronounced. The size of these triangles was relatively large, so that not a simple dipole mode, but higher order modes were excited by the incident light, demonstrating the potential of these methods for imaging also complex field distributions in fine detail.

## Experimental

### Optical set-up

In our femtosecond experiments we used laser irradiation provided by a multi-pass Ti:sapphire amplifier system with a maximum pulse energy of up to 8 mJ, a nominal pulse length of 150 fs, and a center wavelength of 800 nm. For picosecond experiments, the laser beam was coupled out of the amplifier before passing the compressor stage. In this case the pulse length of the laser was measured to be 300 ps (full width at half maximum, FWHM).

**Figure 1:**
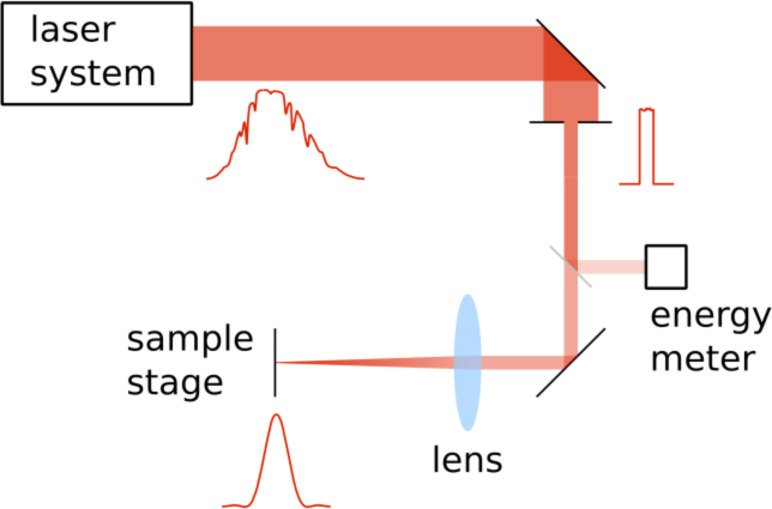
Optical set-up. The sample is mounted on an x–y micrometer stage and can be moved reversibly below a video microscope, which allows to determine the position for irradiation on a micrometer scale. The beam diameter behind the aperture is 2 mm, the focal length of the lens is varied between 200 and 75 mm.

In our measurements, the spatial beam profile of the laser is of vital importance: To gain insight into more than the distribution of the near-field enhancement of a nanoscale object, the absolute enhancement factor has to be measured. In near-field photography, this is commonly done by comparing the threshold of the imaging mechanism (e.g., ablation threshold) without near-field enhancement to one with a scattering nanostructure present during illumination (nanoscale ablation threshold). This route requires the precise knowledge of the fluence distribution of the illuminating laser spot. When a well-defined function describing the energy distribution of the laser spot is known, the determination of the local fluence is reduced to a measurement of the distance from the beam center in combination with a measurement of the total energy of the illuminating laser pulse. For a Gaussian intensity distribution on the sample surface, a simple method to measure the important parameters (the FWHM and the peak intensity *I*_0_) of the distribution has been suggested by J. M. Liu. [27]. Using this method, a substrate with a threshold behavior is illuminated with laser pulses of different total power. The threshold as well as the width of the Gaussian intensity distribution can be directly deduced from the power dependence of the spot size obtained on substrates with an ablation threshold fluence. With a TEM00 mode as the illuminating laser mode and a large lens, the assumption of a Gaussian intensity distribution is justified. In the case of the set-up used in this work, however, the intensity distribution of the laser amplifier system was rather inhomogeneous. Therefore, in order to produce a well-defined intensity distribution on the sample surface, a circular 2 mm aperture was positioned in a region of the beam with nearly constant intensity. Afterwards, the selected beam was focused onto the sample surface (Figure 1).

This situation is described by Fraunhofer diffraction and leads to an Airy-like intensity distribution in the focal plane of the lens [28]:

[1]
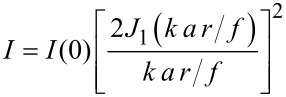


with

[2]
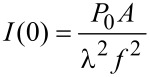


(*J*_1_: first order Bessel function of the first kind; *k* = π/λ: wave vector of the incident irradiation; λ: wavelength of the incident irradiation; *a*: radius of the illuminated aperture; *r*: distance from the intensity maximum on the sample surface; *f*: focal length of the lens; *P*_0_: total irradiation power; *A* = π*r*^2^: area of aperture).

**Figure 2:**
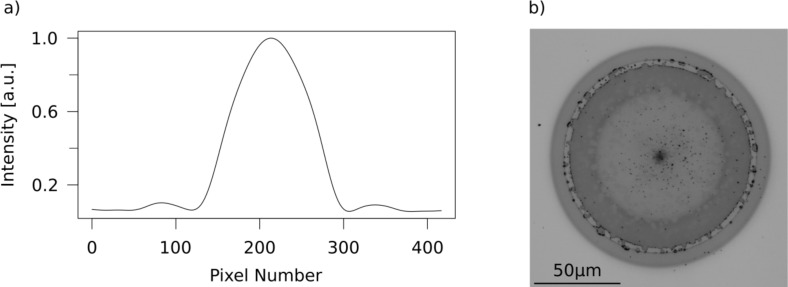
(a) Laser profile at the sample position taken with a CCD camera. Next to the central maximum the first Airy ring is clearly visible. (b) Optical microscope image of a Ge_2_Sb_2_Te_5_ (GST) film, which was irradiated by a single laser pulse (15.5 μJ). In the central region the GST is completely ablated. The dark ring around this region shows were the local fluence was high enough to induce a phase change from the reflecting, crystalline to the amorphous, absorbing state of the GST.

Figure 2a shows a cross section of such an Airy pattern, obtained with a simple CCD camera. For a more exact determination of the profile we have used films of crystalline Ge_2_Sb_2_Te_5_ (GST) as detector material, which has already proven its versatility as a medium for quantitative imaging of fluence distributions earlier [16]. These films were irradiated by pulses with various total energies. Both femtosecond and picosecond pulses induce a phase transition in GST, changing the film from a highly reflecting crystalline into an optically absorbing amorphous state, when the local fluence exceeds a certain threshold fluence *I*_th,GST_. As illustrated in Figure 2b, this leads to a circular amorphous region, whose radius depends on the total energy of the respective pulse. The relation between the threshold fluence and the radius *r*_i_ for a certain total energy *P*_i_ is given by

[3]
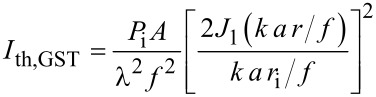


The radii *r*_i_ obtained for the various pulses are determined by an optical microscope. From a fit of these data to Equation 3 the detailed fluence profile can be derived. (Such a fit is shown in Figure S1 in Supporting Information File 1.)

### Sample preparation

**Figure 3:**
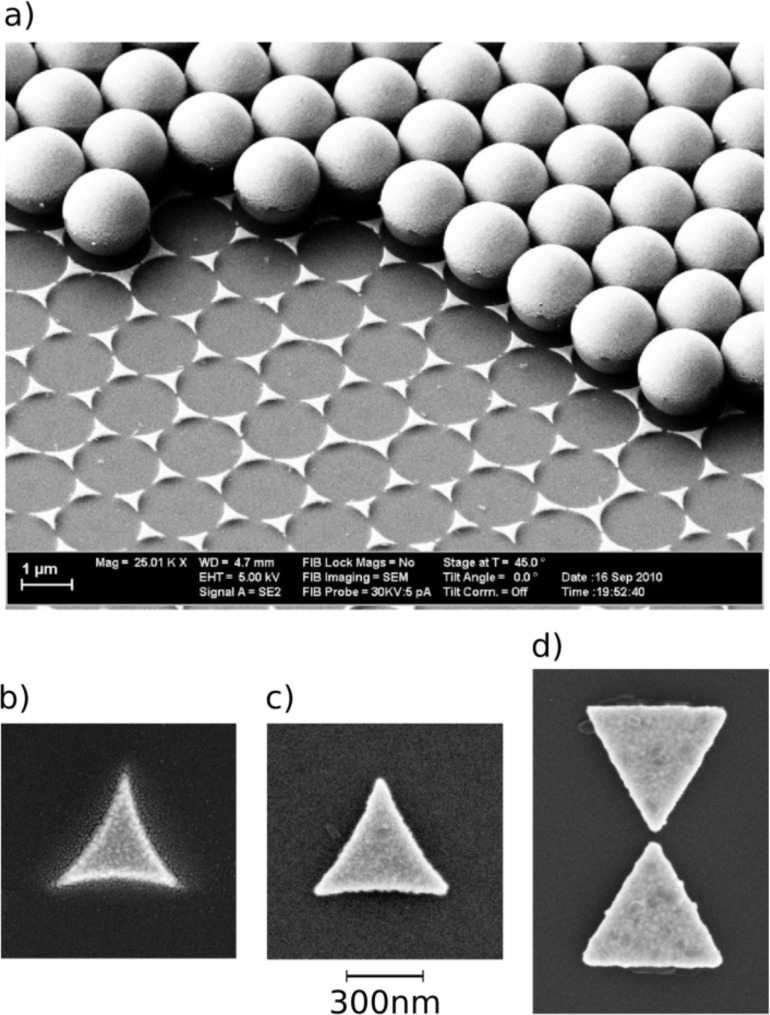
Scanning electron micrographs of different types of triangles used in the course of this work. The triangles in (a) and (b) were prepared by colloid lithography, the triangles in (c) and (d) were prepared by e-beam lithography. In (a) only part of the nanosphere evaporation mask has been removed to illustrate the pattern formation.

**Figure 4:**
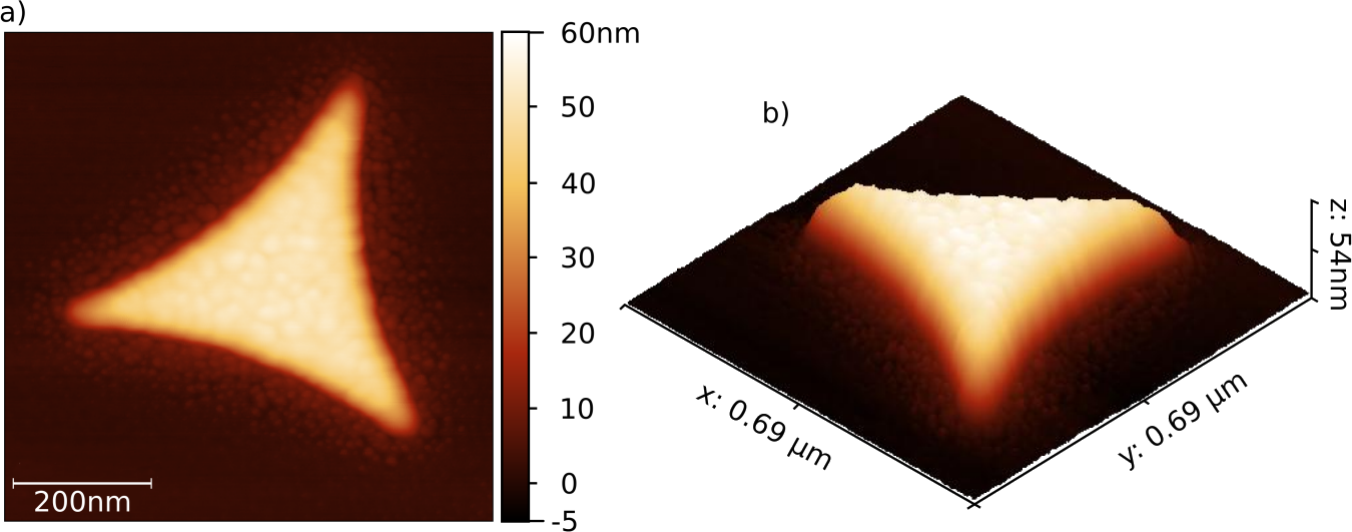
AFM image of a colloid lithography triangle. (a) Top view; (b) 3D image.

**Figure 5:**
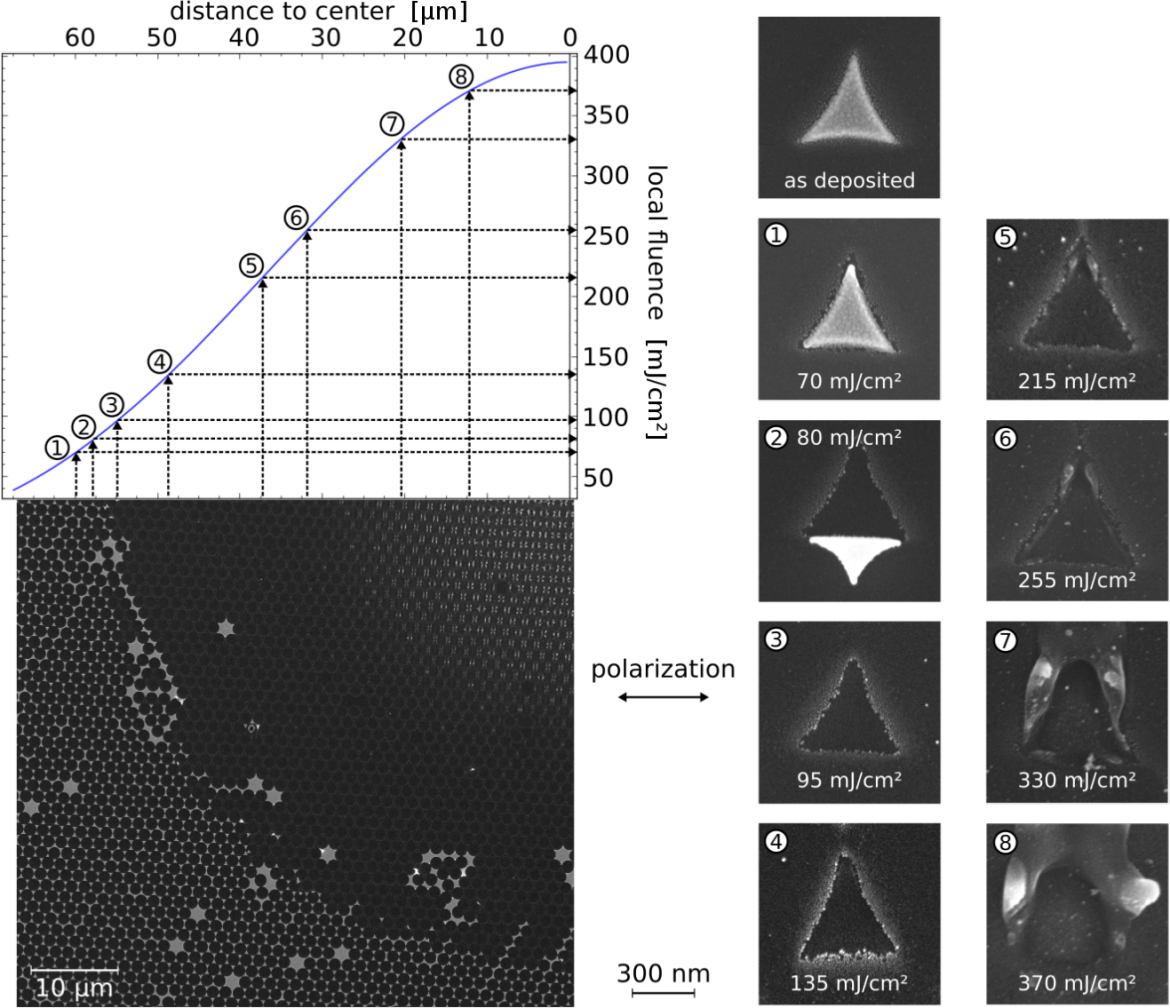
Upper left: Fluence distribution along the laser spot radius as described in the Experimental section. Lower left: SEM picture of one quadrant of an ablation pattern of gold triangles irradiated with a 150 fs laser pulse. The triangles were produced by colloid lithography using spheres with a diameter of 1.55 µm and had a side length of about 530 nm. Right: Detailed blow-ups of triangles and the Si surface in different states of modification. The radial position of each blow-up is marked in the fluence distribution. It has to be noted that the triangles exhibit two different modes, depending on the polarization of the exciting light. Thus two different ablation patterns can be observed. In this figure patterns for a polarization parallel to the edge of the triangle are shown. A comparison of the two different modes can be found in Figure 12.

For the preparation of the plasmonic structures two kinds of nanostructuring methods have been applied, namely colloid lithography and electron beam lithography. The material used was exclusively gold. Figure 3a and Figure 3b present an example for the first and Figure 3c and Figure 3d for the second technique, respectively. In colloid lithography, self-assembled monolayers of meso- or nanoscopic spherical particles are produced by self-organization and subsequently used as an evaporation mask, as described for the first time by Fischer and Zingsheim [29] and Deckman [30]. Since then, the technique has been improved by many groups to yield large arrays of different nanostructures in merely one step [31–36]. The main advantage of colloid lithography is that, while it is relatively easy to implement, periodic arrays of uniform nanostructures can be distributed over large areas (up to a few cm^2^). Furthermore, the triangular structures prepared with this method can have tip radii as small as 5 nm, a size which is difficult to achieve by conventional lithography. On the other hand, in contrast to e-beam lithography samples, the edges of colloid-lithography structures have somewhat inclined walls, and they are surrounded by an ensemble of small solid droplets, both as a result of the evaporation process. These features are clearly visible in Figure 4 as well as in Figure 3b. While the inclined walls may have some influence on the details of the optical near fields, the droplets are negligible in this respect, and they can serve as useful marks for the original position of the triangles when the triangles themselves have been removed by the laser pulse (see, e.g., Figure 5, which will be discussed in detail below). The electron-beam lithography samples were prepared on a silicon (Si) wafer with a native silicon oxide (SiO_2_) layer with a thickness of 2.4 nm (measured by ellipsometry), for the wafers used for colloid lithography the oxide was 1.5 nm thick. Scanning electron microscopy (SEM) and atomic force microscopy (AFM) were used for measuring the dimensions of the plasmonic structures as well as the resulting modifications of these structures and the substrate.

### Simulations

We used the program package “FDTD Solutions” by Lumerical Inc. to simulate the spatial distribution of the electric field of a triangle and the energy dissipated during its illumination. To get realistic results, an AFM image of a gold triangle like the one in Figure 4 was used as a basis for generating the geometry of the simulation. This method allows for taking into account the details of the shape of a triangle (the curvature of the corners, the profile of the edges and the surface roughness), which have significant influence on the coupling between the triangle and the incident light wave.

**Figure 6:**
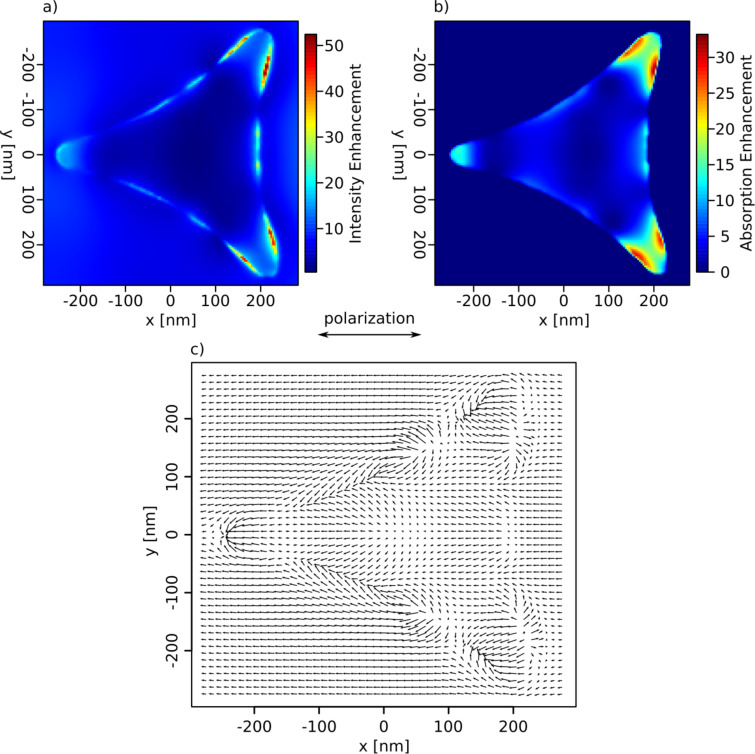
(a) Calculated field intensity enhancement, (b) dissipation, and (c) field distribution for a nanotriangle like in Figure 4. (The model structure on which the simulation was based is depicted in Figure S2 in Supporting Information File 1). The polarization of the incident laser radiation was here horizontal. The simulation volume was 1 × 1 × 1.2 µm^3^ with "perfectly matched layer" boundary conditions. The meshing was set to automatic mode far from the triangle and a manually refined mesh with a cell size of 1.3 × 1.3 × 0.5 nm^3^ was added around the particle.

The substrate of the triangle used for the calculations was chosen to consist of a 1.5 nm thick SiO_2_ layer on top of bulk Si. In Figure 6, the results of such a simulation are shown. Figure 6a depicts the field intensity around the triangle. The absolute values denote the intensity enhancement compared to the incident intensity (averaged over the whole height of the triangle (40 nm)). In Figure 6b the energy, which is dissipated inside the triangle, is shown. For this graph the results of two simulations were combined: The values gathered from a simulation describing a single triangle on a silicon surface were divided by the values of a simulation with the same parameters, describing a continuous gold film. This results in a factor, which depicts the local enhancement of the dissipated energy, as it is caused by the triangle. As before, the values were averaged over the triangle height. Figure 6c displays the electrical field vectors at 10 nm height inside the triangle.

## Results and Discussion

### Femtosecond laser ablation

#### General features

We first give an overview over the general features of femtosecond plasmonic ablation. Figure 5 shows an SEM micrograph of a typical femtosecond ablation site on a sample, which consists of gold nano-triangles prepared by colloid lithography. The plasmonic structures were irradiated with a single femtosecond laser pulse with a total energy of 30 μJ. The irradiation intensity increases towards the center of the irradiated spot, and, since the spot profile is known, the local fluence can be determined by measuring the distance from the ablation center. The details shown to the right of the overview visualize the different stages of the removal/ablation process as a function of the local fluence. The *calculated* near-field distribution for similar triangles has been shown in Figure 6a. Looking at the SEM micrographs, several thresholds can be distinguished for single pulse femtosecond irradiation. Starting at low intensities, first a modification of the triangles is observed at those tips, where the field enhancement is largest. For somewhat higher intensities, the triangles are removed from the surface. In some cases, the complete triangle is tilted from its original position (see frame (2) of Figure 5) or found somewhere else on the sample surface. Note that, in contrast to picosecond (see below) or nanosecond [37] irradiation, the modification of the triangles is only slight for fluences below the removal threshold or even for redeposited triangles. Triangles in more advanced states of melting could not be found for femtosecond ablation.

For the particles presented in Figure 5, the removal threshold is followed by a region where no modification of the underlying surface is discernible by SEM or AFM measurements. Above a certain *incident* local fluence *I*(*x*), however, which we denote as 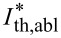, an ablation of the substrate's surface is visible. (The asterisk indicates that 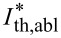 is smaller than the ablation threshold of the bare substrate, *I*_th,abl_, due to the local field enhancement factor FE: 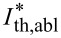
·FE = *I*_th,abl_). The fluence associated with this ablation threshold of the plasmonic structures is strongly dependent on the particle size and shape and can be higher, the same or even lower than the removal threshold. Above the ablation threshold 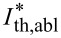, the surface modification takes the form of material removal as well as droplets of apparently molten and resolidified material [38]. For even higher intensities, the ablated region increases, with the size of the accompanying droplets increasing accordingly. Eventually, the substrate is modified everywhere also between the positions of the plasmonic structures. We will focus here on the intensity range around the ablation threshold 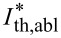. The higher intensities are beyond the scope of this article and will be considered elsewhere.

#### Determination of the ablation threshold

**Figure 7:**
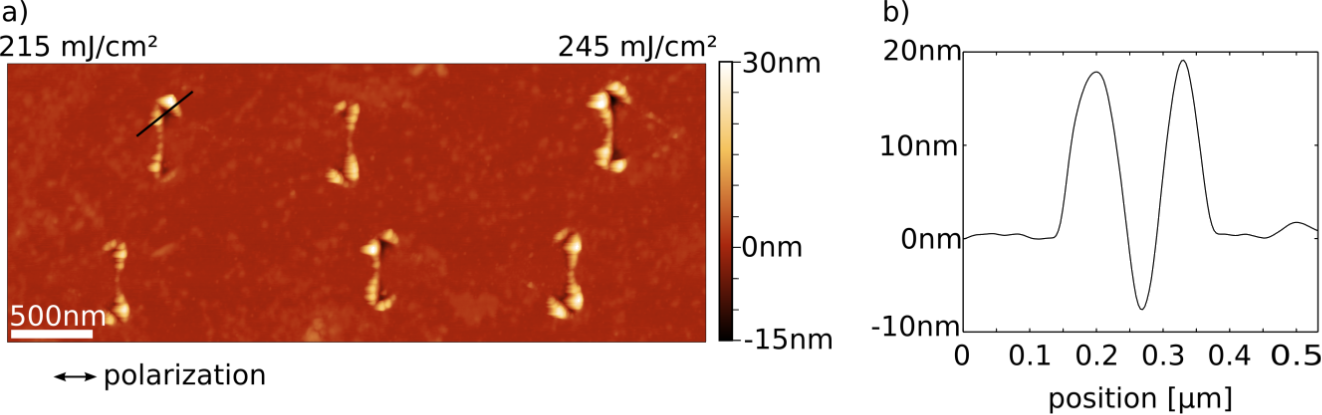
(a) AFM image of a Si surface with ablation holes, generated by irradiation of colloid lithography triangles with a single femtosecond laser pulse. (b) height profile of one of the holes taken along the black line in (a). The arrow indicates the polarization of the laser light. The incident intensity increases to the right from 215 to 245 mJ/cm^2^. Gold droplets resulting from sample preparation and ablation have been removed with aqua regia. Here, the volume of the material deposited in the rim appears to be distinctly larger than the volume of the crater hole. This is due to a change in composition (the rim consists mostly of silicon oxide), and partly also due to the fact that the AFM tip is not sharp enough to trace out all details of the crater profile.

**Figure 8:**
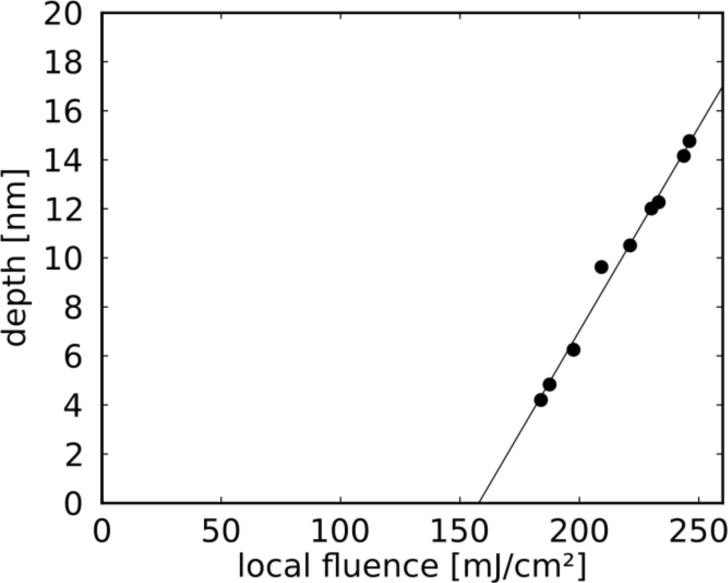
Hole depth (as determined from profiles such as in Figure 7b, measured with respect to the height of the flat surface) as a function of the incident local fluence.

A first estimate for the ablation threshold 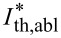 in the near field of the plasmonic triangles can already be obtained from Figure 5, where the frames for 135 and 215 mJ/cm^2^ are below and above the threshold, respectively. In order to determine the value of 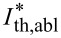 more accurately, we have taken AFM scans, such as the one shown in Figure 7a, which allow one to measure the depth of the ablation holes. An example for the profile of an ablation crater at an incident (local) fluence of 220 mJ/cm^2^ is given in Figure 7b. The depth of such holes has been measured for various positions across the irradiated spot, i.e., as a function of the incident fluence *I*(*x*). The result is plotted in Figure 8. Assuming, for simplicity, a linear relationship between depth and (*I*(*x*) − 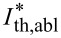) in the intensity regime close to threshold, we obtain a threshold value for 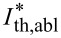 of 160 mJ/cm^2^.

This value can be compared to the ablation threshold of bare Si, *I*_th,abl_, which according to the literature is close to 400 mJ/cm^2^ for single laser pulses at similar wave length and pulse duration [24–26]. From the ratio of the two thresholds one arrives at an intensity enhancement of 2.5 at the two triangle tips which cause the ablation holes in Figure 7. This result has to be considered with care, however, because the threshold value for the ablation of bare Si appears to vary between different experiments and also depends on assumptions about the beam profile [25]. We have therefore determined the ablation threshold for bulk Si with the same set-up and under the same conditions as for the plasmonic structures described above.

**Figure 9:**
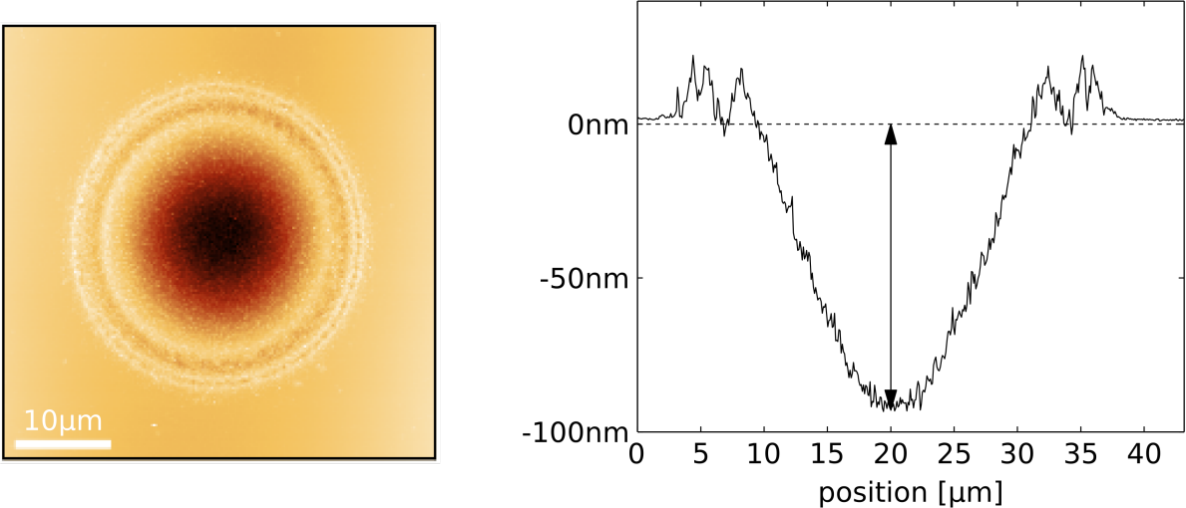
AFM image and height profile of an ablation hole generated by far-field ablation of bare Si (same wafer as in Figure 7). Note that the beam profile in this case is again described by an Airy distribution, comparable with the irradiation of the nanostructures.

**Figure 10:**
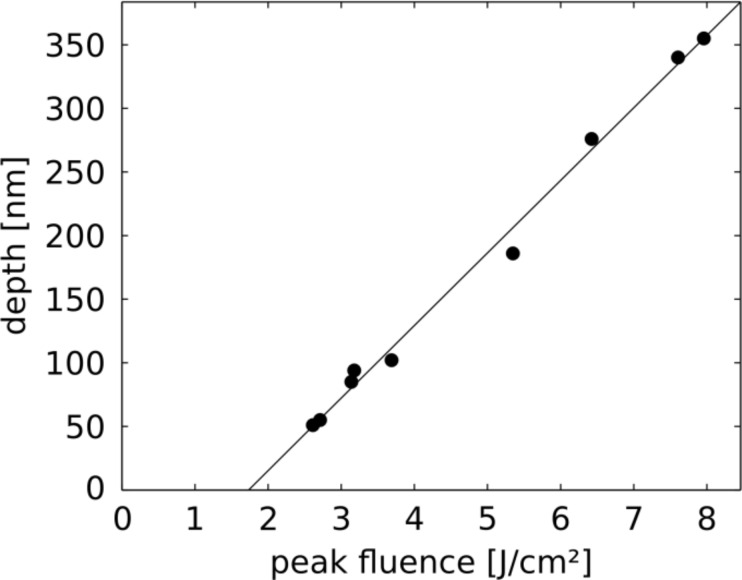
Depth of holes in Si, generated by far-field ablation, as a function of the peak fluence.

Figure 9 shows an AFM image and the height profile of the ablation hole for a peak fluence of 3.2 J/cm^2^. Carrying out this experiment for different total energies of the incident laser pulse provides the depth of the holes as a function of the fluence, as depicted in Figure 10. The extrapolation yields a threshold value of 1800 mJ/cm^2^, nearly a factor of five higher than the literature value mentioned above. The absolute values of the hole depths are in close agreement with results of Nedyalkov et al. for fluences above 2 J/cm^2^ [1]. The apparent difference in the threshold values can be ascribed to two regimes with different ablation mechanisms: whereas at low fluences non-thermal ablation dominates, the regime above about 2 J/cm^2^ is governed by thermal effects, including explosive boiling, which give rise to distinctly higher ablation rates [39]. Since the craters generated by ablation in the near field of the nano-triangles resemble the craters on bulk Si generated by far-field illumination in the high-intensity regime, we compare the extrapolated values from the linear fits in Figures 8 and 10, namely 160 and 1800 mJ/cm^2^ for near-field and far-field illuminations, respectively. Under this assumption the resulting intensity enhancement factor is about 10, a factor of two to five below the results of the simulations in Figure 6a and Figure 12 (shown below).

#### Shape dependence

To demonstrate the dependence of both field distribution and field enhancement on the details of the shape of a nanostructure, two similar types of gold nano-triangles with differently shaped edges have been used: For one type the edges were slightly curved inwards to mimic the shape of triangles prepared by nanosphere lithography, while for the second type the edges were straight, like the triangles simulated in early DDA calculations [21]. The structures in this case were prepared by e-beam lithography. Note that, even though the shape of the triangles prepared by colloid lithography and the structures with concave edges presented here seem similar when looked at from above, the triangles prepared by e-beam lithography have much steeper ridges due to the different preparation mechanism.

**Figure 11:**
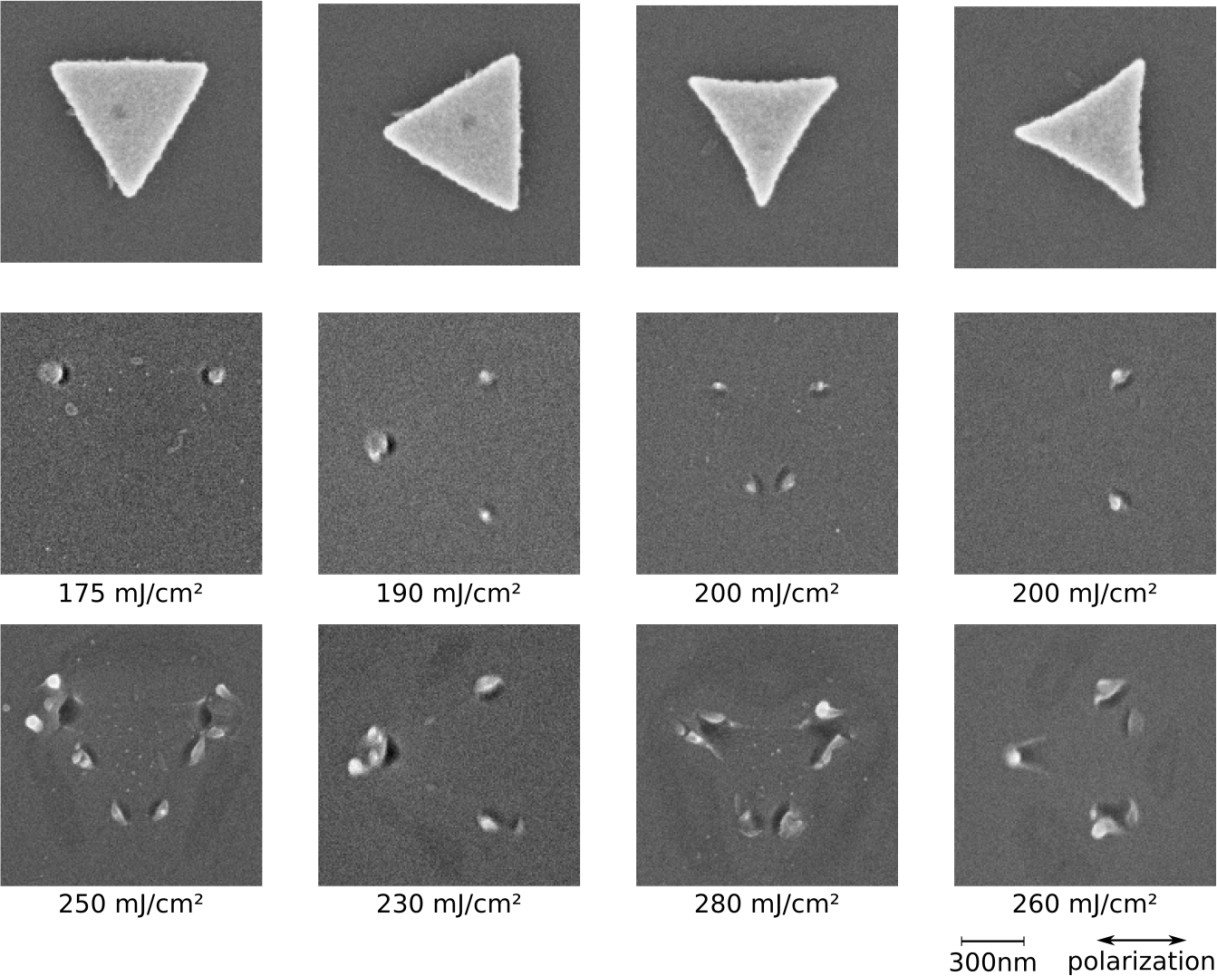
SEM micrographs of two different types of nanotriangles prepared by electron beam lithography, and their corresponding ablation patterns for two orientations with respect to the polarization of the incident laser light. The local fluence is indicated below each frame.

Figure 11 shows SEM micrographs of the triangles and their corresponding ablation patterns for two different orientations with respect to the polarization of the incident laser pulse, and for the two differently shaped edges. Comparing the ablation patterns for both structures and orientations, the ablation distributions are evidently different. Even though the structures are of comparable size and their basic geometry is the same, the exact shape of the edges of the triangles has a major effect on the ablation distribution. The triangles with the concave edges, which resemble the colloid lithography triangles, exhibit an ablation pattern similar to the pattern of those (Figures 5 and 7), as expected. The straight-edged triangles, on the other hand, show an ablation pattern much closer to a simpler (dipolar-like) expectation: Slightly above the ablation threshold, ablation can only be found below the tips of the triangles which are oriented in the direction of the polarization. Only for higher local fluences, a more complex ablation distribution is revealed. The threshold values for plasmon-induced ablation 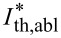 of these triangles were a factor of two lower than for the concave triangles.

**Figure 12:**
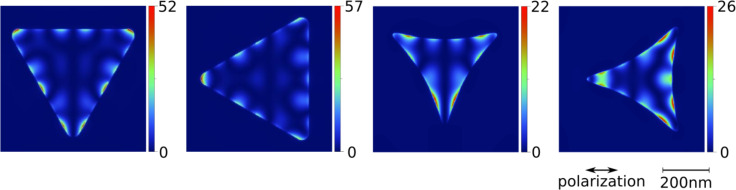
FDTD calculations for the structures presented in Figure 11. The field intensity enhancement was extracted in a plane between the SiO_2_ layer (thickness: 2.4 nm) and the Si substrate below the triangle. The simulation volume was 3 × 3 × 6 µm^3^ with "perfectly matched layer" boundary conditions. The meshing was set to automatic mode far from the triangle, and a manually refined mesh with a cell size of 2 × 2 × 0.5 nm^3^ was added around the particle.

FDTD simulations for both types of triangles can be found in Figure 12. Note that, in contrast to the simulation in Figure 6, the simulation in Figure 12 is based on a more idealized geometric structure with steeper ridges. This is justified, as the ridges of the structures are less rounded down due to the preparation process. Again, the distribution of the local field enhancement matches the distribution of the ablation pattern quite well. Comparing the calculated patterns with the ablation patterns in Figure 11, one can see that the regions with highest calculated intensities already show ablation for local fluences slightly above the ablation threshold (Figure 11, second row). Ablation in the regions with lower calculated intensity becomes visible as the local fluence increases (Figure 11, third row). The comparison also shows that the calculated enhancement factors for the straight-edged triangles are roughly a factor of two higher than for the concave triangles, which goes in line with the ratio of the corresponding ablation thresholds mentioned above. The absolute enhancement values determined from experiment, however, are again distinctly lower than the FDTD results.

#### Minimum structure size

As mentioned in the introduction, plasmon-mediated ablation is an interesting method for optical patterning of surfaces far below the diffraction limit as well as for imaging optical near fields. The ablation hole shown as an example in the profile of Figure 7b has a depth of 7 nm and a width of 40 nm. The smallest holes we obtained in Si substrates so far, observed for plasmonic triangles of 70 nm side length, were 5–10 nm wide [40]. One might ask whether this is a fundamental limit, or whether by ablation of Si one could image even smaller details of the optical near-field distribution.

**Figure 13:**
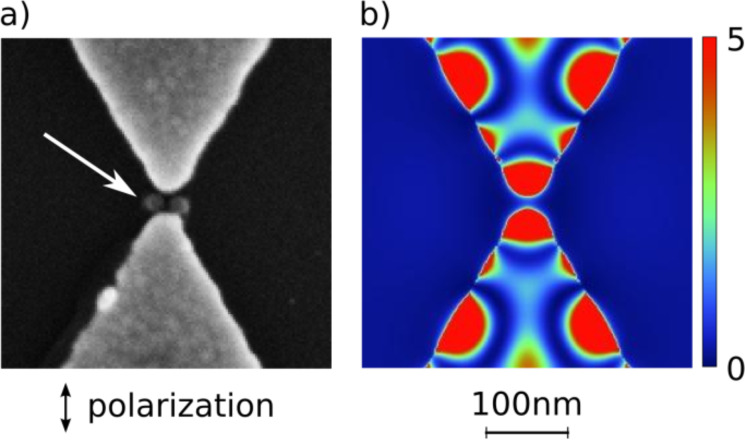
(a) SEM micrograph and (b) calculated field distribution for the center region of a bow-tie antenna with a distance of 30 nm. The structure has been irradiated with a 150 fs laser pulse (wavelength: 800 nm) with a local fluence of 90 mJ/cm^2^. The arrow points towards the region in the feed gap where ablation of the Si has taken place. The color scale in (b) has been chosen such to show the field distribution in the region between the triangles.

In order to address this problem we have investigated so-called bow-tie antennas, consisting of two triangles as shown in Figure 3d. Figure 13a shows the region between such a bow-tie antenna with a gap distance of 30 nm, after it has been irradiated with a local fluence of 90 mJ/cm^2^. Figure 13b represents the calculated near-field intensity distribution of this configuration. The color scale for the distribution has been adjusted to visualize the intensity distribution between the triangles. One can see in Figure 13a that distinct nano-ablation between the two triangle tips has taken place (arrow in Figure 13a), potentially even with fine structure. The resolution of the SEM image however does not allow one to unambiguously determine the shape of the ablation hole. Note that in this case the incident fluence was even below the triangle removal threshold. In the same region of Figure 13b the calculated enhancement factor is on the order of one, thus it is quite unlikely that the enhanced local fluence would reach the fluence *I*_th,abl_ needed to ablate the underlying Si substrate, which is one order of magnitude higher.

This observation suggests that some broadening mechanism is effective, which disperses the energy deposited by the plasmonic near fields. Such a mechanism could not only explain the ablation in between the triangles in Figure 13a, but might also be one of the reasons for the significantly lower enhancement factors found in all our ablation experiments when compared to the intensity enhancement calculated by FDTD. When dissipated over a wider region, the overall effect of the near-field enhancement is diminished by a factor given by the ratio of the volume with an intense near field and the dissipation volume. A possible mechanism for the broadening has been outlined by Kolloch [40] based on the fast diffusion of the charge carriers in the light-induced electron–hole plasma in the silicon. A broadening of the ablated hole structure compared to the calculated intensity distribution has also been observed recently by Robitaille et al. [26] for Au *nanorods* on Si when irradiated with femtosecond pulses, and the quantitative agreement with a carrier-diffusion-based model is reported.

#### Picosecond laser melting

In contrast to the femtosecond irradiation studies presented above, thermal diffusion is the dominating factor when ablation is done with laser pulses with a duration in the higher picosecond and nanosecond regime [41]. For near-field ablation this leads to a significant decrease in imaging resolution and renders this timescale useless for nanoscale material processing of the substrate. The resulting modifications of the plasmonic structure itself, however, are interesting both from a technical as well as a fundamental point of view. For fundamental considerations, the dominance of thermal diffusion leads to a simplification in the distribution of the energy provided by the incoming laser pulse. As the only discernible process of material modification is melting, the near-field distribution can simply be considered acting as a heat source. This reduces the complexity of the calculation and the simulation of the thermal and mechanical processes after irradiation considerably. From a technical point of view, the system provides a spatially confined heat source, which is only limited by the thermal diffusion length given by the pulse duration of the illuminating laser.

#### General features

**Figure 14:**
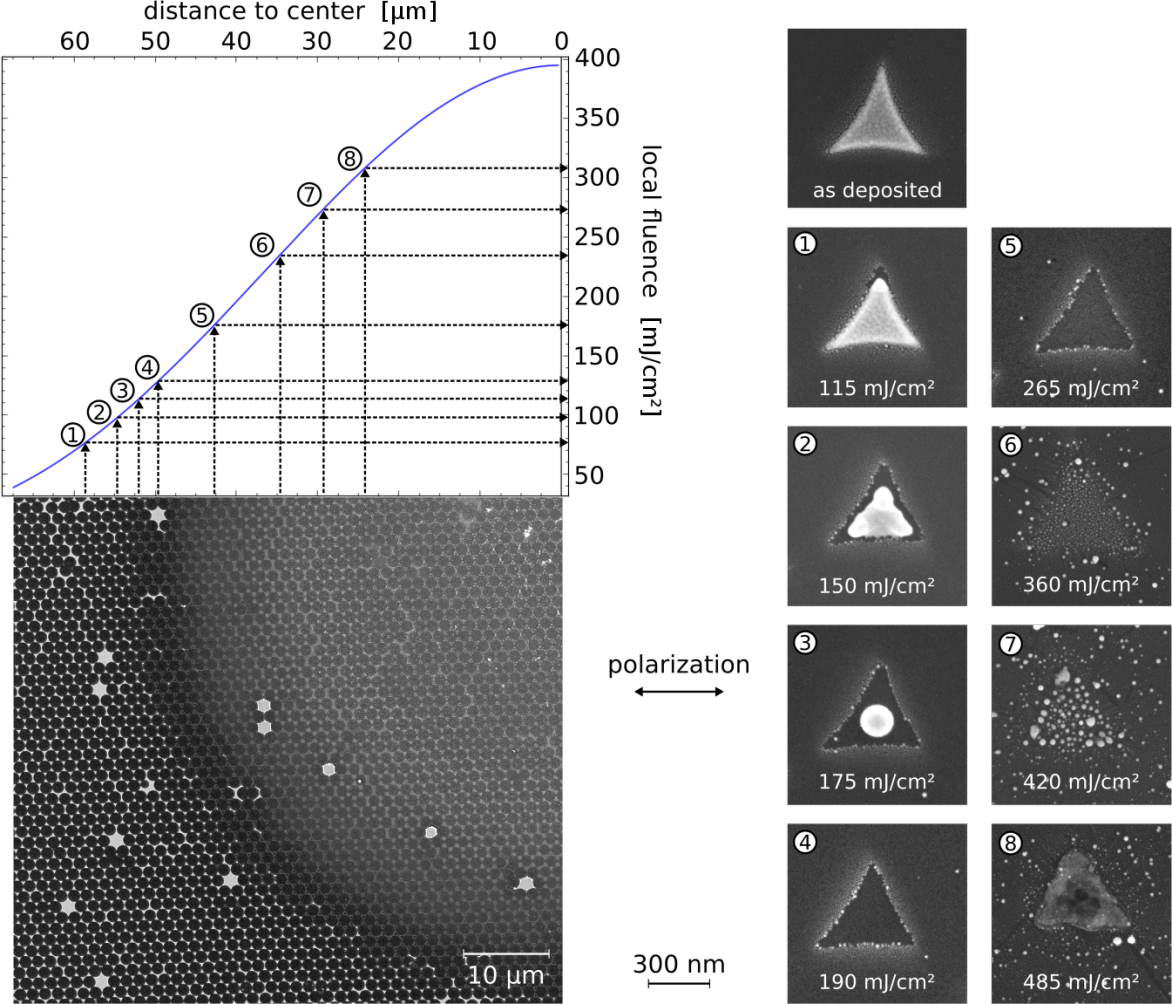
Evolution of laser-molten gold triangles (thickness: 40 nm, side length: 530 nm, prepared by colloid lithography with 1.55 µm diameter spheres) for increasing laser fluence in equivalent presentation as Figure 5: Upper left: Fluence distribution along the spot radius. Lower left: SEM micrograph of one quadrant of an ablation spot irradiated with a ps laser pulse (wavelength: 800 nm, FWHM: 300 ps). Right: Details at different levels of incident laser fluence. The local fluence is indicated in each frame.

Figure 14 gives an overview of the different stages of the picosecond melting/ablation for gold triangles with a side length of 530 nm. The plasmonic structures used for the melting experiments were again produced by colloid lithography.

For triangles just above the modification threshold (Figure 14, frame 1), the tip with the highest local plasmon intensity has been deformed into a spherical droplet, while the tips with low near-field enhancement have not been altered. Additionally, the surface texture of the triangles in the modified region has changed. While the unmodified regions have a roughness comparable to the deposited gold film, the surface structure in the regions, which show a change due to the laser radiation, is much smoother. This phenomenon, which is ascribed to melting, can also be used for the determination of the melting threshold of a homogeneous film (see below). With increasing local fluence, larger parts of the triangles are deformed, until finally a spherical smooth particle has developed at the position of the original triangle.

Above a local fluence threshold of 180 mJ/cm^2^, no gold particles comparable to the size of the original triangles can be found anywhere on the substrate. For even larger intensities, however, a formation of droplets in the former triangle positions and in between these positions is observed. The droplets consist of gold, since they can be removed by aqua regia. As in the previous chapter, the laser-induced modifications seen at high intensities well above the melting threshold will be discussed elsewhere.

#### Melting threshold

**Figure 15:**
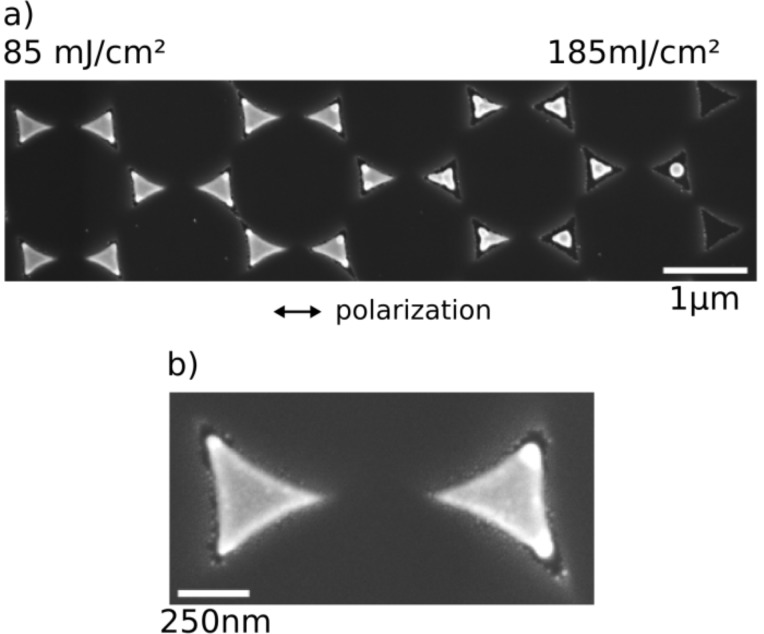
(a) SEM micrograph of colloid lithography nanotriangles irradiated with a 300 ps laser pulse (wavelength: 800 nm) with high gradient to show all typical states of the melting process in one image. The local fluence increases to the right from 85 to 185 mJ/cm^2^. (b) Detail from (a). The scale bars are 1 µm and 250 nm, respectively, the polarization of the incident light is horizontal.

As shown in Figure 15, apparently also the local melting allows one to image the regions of high near-field enhancement. Due to thermal diffusion the spatial resolution reached here is lower than for local ablation, but the overall patterns of the intensity distribution turn out to be quite similar. In spite of the obvious broadening taking place during local melting, also in this case an estimate for the plasmonic field enhancement can be obtained. We have used the same strategy as in the previous chapter: When the volume of the affected zone is determined as a function of the local intensity, a threshold value for the onset of melting can be derived. This threshold for the plasmon-induced melting is then compared with the threshold for the melting of the homogeneous system, i.e., an unstructured Au film, under far-field illumination.

**Figure 16:**
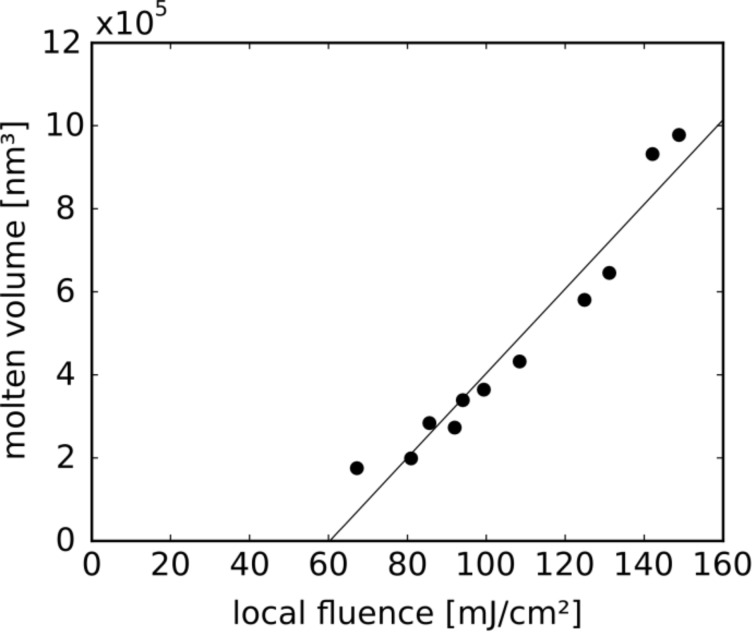
Molten volume at the triangle tips vs local laser fluence.

Figure 15a displays an SEM image of partly molten triangles in an intensity regime between 85 and 185 mJ/cm^2^, and Figure 15b shows a blow-up. Note that the intensity gradient used in this irradiation was so steep that the right triangle in Figure 15b, located less than 1 µm from the left one, exhibits considerably larger molten volumes at the tips. Plotting the molten volume, as determined from the SEM images, versus the incident intensity leads to the graph in Figure 16, from which a plasmon-enhanced threshold value 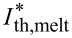 of 60 mJ/cm^2^ for the melting of the tips can be derived.

**Figure 17:**
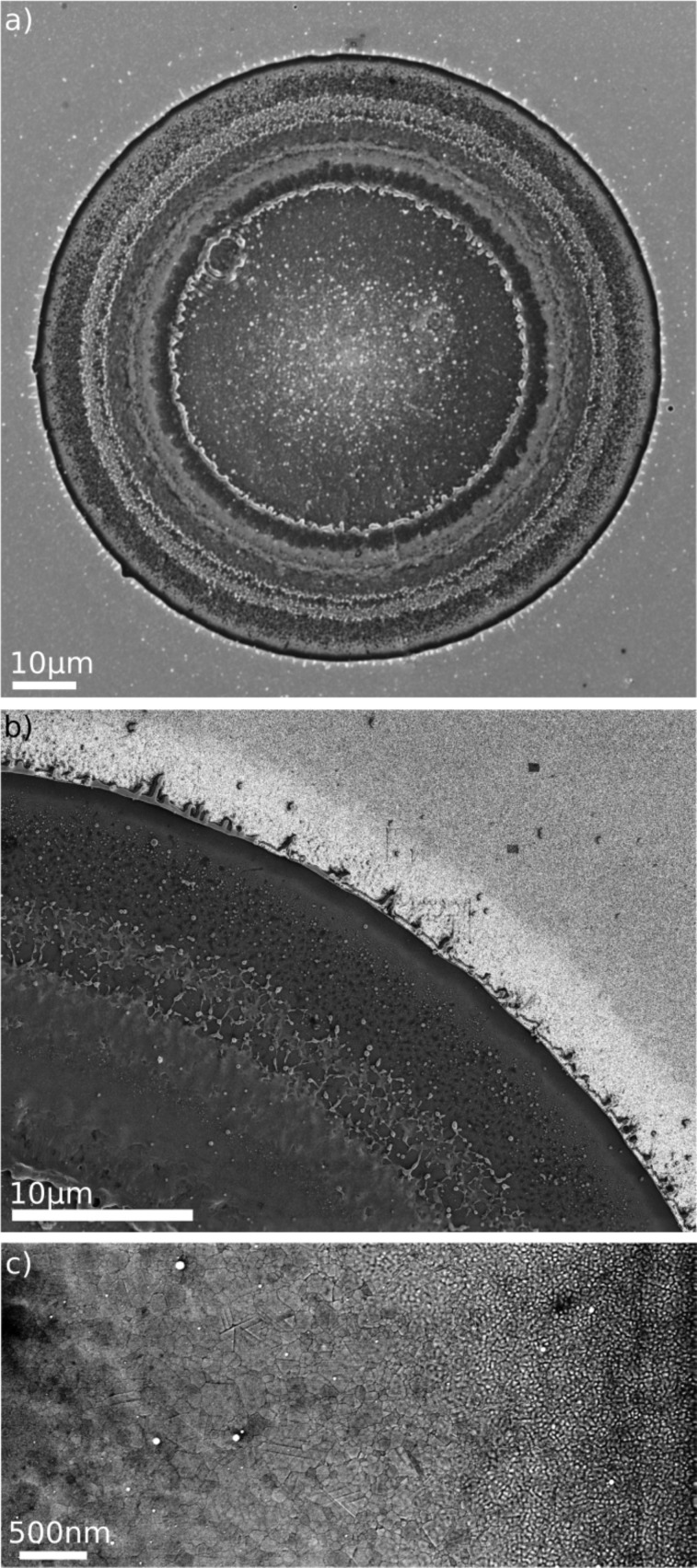
SEM micrographs of a 40 nm Au film on Si, after exposition to a 300 ps laser pulse at 800 nm wavelength. (a) Overview over the ablation spot, (b) and (c) blow-ups of the transition region with two different magnifications.

For the determination of the melting threshold of a homogeneous Au film, which was also irradiated with a single 300 ps laser pulse, we consider Figure 17. The Si substrate and the film thickness were the same as in Figure 15. Figure 17a shows an overview over the whole irradiated spot and Figure 17b shows a magnified section, in which the contrast has been raised. The border between the light and dark grey region in the upper right corner of Figure 17b can be identified as the transition from the molten to the non-molten region. An even larger magnification (Figure 17c) reveals the change in topography: The grainy structure to the right is characteristic of the as-evaporated Au films, which in our samples have a typical roughness of 5 nm. When the films are molten and solidify quickly on a nanosecond scale, they become smoother and exhibit grain boundary structures. (For larger melting times the films would start to dewet and would develop holes [42].) The melting threshold, *I*_th,melt_, calculated from Figure 17 and the corresponding Airy intensity profile, amounts to 540 mJ/cm^2^. When comparing this to the threshold value for plasmon-assisted melting 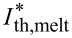, one arrives at an enhancement of 9 ± 2 due to the plasmonic resonance in the triangles.

The enhancement derived from local melting is in good agreement with the value of 10 obtained by the ablation technique from Figure 8 and Figure 10. We would like to point out, however, that this might be a coincidence. In the case of ablation the relevant fields for generating holes in the Si substrates are located *outside* the plasmonic structures, whereas the plasmon-induced melting takes place due to dissipation *inside* the structures.

#### Mechanism

All the findings described above can be explained by ultrafast heating and subsequent melting of the plasmonic nanostructures. Assuming that the heat is produced in the area of the highest near-field intensity during the laser pulse, the comparatively short thermal diffusion length leads to strong thermal gradients with a length scale that is below the dimensions of the illuminated particle (thermal diffusion length: *l*_D_ = 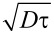 ≈ 200 nm with thermal diffusivity of gold *D* = κ/(*c*_p_·ρ) = 1.27·10^−4^ m^2^·s^−1^ and laser pulse length τ = 300 ps; κ: thermal conductivity; *c*_p_: specific heat capacity; ρ: mass density). For higher intensities, larger parts of the triangles melt. In analogous to fs pulses there is an intensity regime where the nanostructures are removed by the laser irradiation without causing major damage on the Si substrate surface. The removal process of these molten structures is different, however, from the removal of (nearly intact) triangles by fs pulses. It has been described for the case of nanosecond pulses by Habenicht et al. [37]. Basically, the potential energy that is stored in a flat triangle as compared to the round droplet form on a non-wettable surface is transferred into kinetic energy that launches the particle from the surface.

## Conclusion

The results reported here provide a test bed for the determination of near fields of plasmonic nanostructures, based on the one hand on an experimental mapping of the regions of pronounced field enhancement, on the other hand on FDTD simulations. When comparing the field-enhancement distributions obtained from experiment with the calculations, one finds excellent agreement concerning the spatial intensity patterns, whereas calculated enhancement factors are typically somewhat larger than the experimental ones, yet of the same magnitude. This sheds light on the reliability of FDTD simulations, for which it is apparently crucial that all the details of the plasmonic structures are taken into account, if one aims at a quantitative prediction of the near fields. Although a similar finding has already been reported earlier, this work provides the first quantitative information for the relatively complex higher-order plasmon modes of the triangular structures used here. From this knowledge one can draw conclusions on how trustworthy simulations of even more complicated plasmonic structures, e.g., for the design of sophisticated optical antennas, will be. The method described here should also be applicable for higher order polarizations, like azimuthally or radially polarized laser beams, as they have been used to excite plasmon modes in triangular nanostructures in a confocal microscope [43].

As our study has further shown, the minimum feature size of ablation patterns in Si is limited by a broadening process which is probably related to the diffusion of light-induced charge carriers, restricting structure formation by optical near fields in this material to feature sizes of around 10 nm. Yet nanopatterning by near-field ablation with femtosecond pulses and the related formation of a nanoscale plasma appears as a novel route for certain applications in biology and medicine, where a chemical patterning of a substrate on scales well below optical wavelengths is of interest. The strong thermal gradients in triangles irradiated with *picosecond l*aser pulses might also be interesting for biological applications. Plasmonic particles heated by laser pulses have been discussed to be used as "nanostoves" for biological tissue in biosensors or for cancer thermotherapy [44–45]. They may also lend themselves as sources of local heat gradients in nano thermoelectronic devices [46]. In addition to the spherical particles described in literature, the selective heating of single tips of the plasmonic triangle would make it possible to compare heating effects under otherwise identical conditions on a very small scale.

## Supporting Information

File 1 Line-fit and triangle model.
