# Supplementary material for: Mapping of plasmonic resonances in nanotriangles
Source: Beilstein J Nanotechnol. 2013 Sep 30;4:588–602. doi: 10.3762/bjnano.4.66 (PMC3817793; doi:10.3762/bjnano.4.66)
Supplement: File 1 — Line-fit and triangle model. [file Beilstein_J_Nanotechnol-04-588-s001.pdf]

Supporting Information

for

Mapping of plasmonic resonances in nanotriangles

Simon Dickreuter, Julia Gleixner, Andreas Kolloch, Johannes Boneberg, Elke Scheer and Paul Leiderer*

Address: Physics Department, University of Konstanz, Universitätsstraße 10, 78464 Konstanz, Germany

Email: Paul Leiderer* - paul.leiderer@uni-konstanz.de

* Corresponding author

Line-fit and triangle model

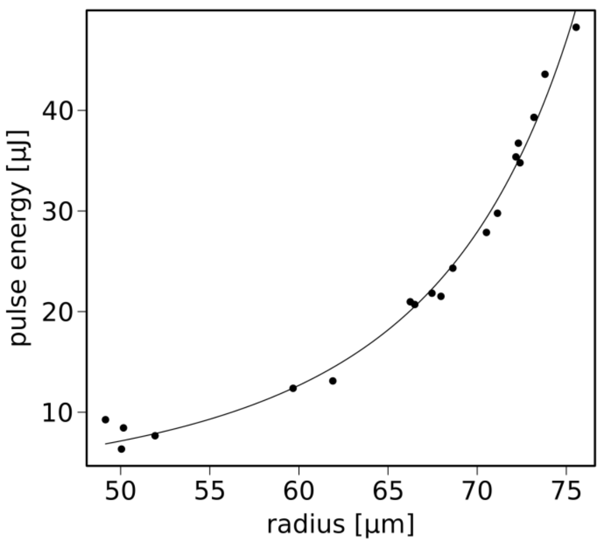

Figure S1: Relationship between the incident laser pulse energy and the radii of the circular amorphous regions in GST, as shown in Figure 2b. The line is a fit according to Equation 3.

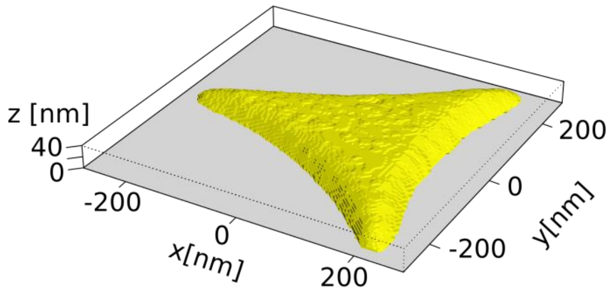

Figure S2: Model for the triangles produced by colloid lithography, on which the simulations displayed in Figure 6 were carried out. It was obtained from the topography data of an AFM image, which were imported into Lumerical. Thus the curvature of the sides as well as the inclination of the walls and the surface roughness of a real nanotriangle are taken into account. These modifications compared to an idealized structure partly shift and partly broaden the plasmon resonance and thus can be responsible for a decrease of the field enhancement in the hotspots.
